# Generation of an *E. coli* platform strain for improved sucrose utilization using adaptive laboratory evolution

**DOI:** 10.1186/s12934-019-1165-2

**Published:** 2019-06-29

**Authors:** Elsayed T. Mohamed, Hemanshu Mundhada, Jenny Landberg, Isaac Cann, Roderick I. Mackie, Alex Toftgaard Nielsen, Markus J. Herrgård, Adam M. Feist

**Affiliations:** 1 Novo Nordisk Foundation Center for Biosustainability, Technical University of Denmark, Building 220, Kemitorvet, Lyngby, 2800 Kgs Denmark; 2 Department of Animal Sciences, Institute for Genomic Biology and Energy Biosciences Institute, University of Illinois, Urbana, IL 61801 USA; 3 Department of Bioengineering, University of California, 9500 Gilman Drive La Jolla, San Diego, CA 92093 USA

**Keywords:** *Escherichia coli*, Renewable feedstocks, Sucrose, Adaptive laboratory evolution, Platform strains

## Abstract

**Background:**

Sucrose is an attractive industrial carbon source due to its abundance and the fact that it can be cheaply generated from sources such as sugarcane. However, only a few characterized *Escherichia coli* strains are able to metabolize sucrose, and those that can are typically slow growing or pathogenic strains.

**Methods:**

To generate a platform strain capable of efficiently utilizing sucrose with a high growth rate, adaptive laboratory evolution (ALE) was utilized to evolve engineered *E. coli* K-12 MG1655 strains containing the sucrose utilizing *csc* genes (*cscB*, *cscK*, *cscA*) alongside the native sucrose consuming *E. coli* W.

**Results:**

Evolved K-12 clones displayed an increase in growth and sucrose uptake rates of 1.72- and 1.40-fold on sugarcane juice as compared to the original engineered strains, respectively, while *E. coli* W clones showed a 1.4-fold increase in sucrose uptake rate without a significant increase in growth rate. Whole genome sequencing of evolved clones and populations revealed that two genetic regions were frequently mutated in the K-12 strains; the global transcription regulatory genes *rpoB* and *rpoC,* and the metabolic region related to a pyrimidine biosynthetic deficiency in K-12 attributed to *pyrE* expression. These two mutated regions have been characterized to confer a similar benefit when glucose is the main carbon source, and reverse engineering revealed the same causal advantages on M9 sucrose. Additionally, the most prevalent mutation found in the evolved *E. coli* W lineages was the inactivation of the *cscR* gene, the transcriptional repression of sucrose uptake genes.

**Conclusion:**

The generated K-12 and W platform strains, and the specific sets of mutations that enable their phenotypes, are available as valuable tools for sucrose-based industrial bioproduction in the facile *E. coli* chassis.

**Electronic supplementary material:**

The online version of this article (10.1186/s12934-019-1165-2) contains supplementary material, which is available to authorized users.

## Introduction

There is a significant interest in the utilization of renewable carbon feedstocks for bioprocesses due to both environmental and economic factors. Sucrose from sugarcane can serve as a renewable carbon source as it originates from a cheap abundant feedstock source which can lower carbon source costs [1, 2], and it can be readily utilized by biological systems in fermentation processes either from the raw source as sugarcane juice [3] or from the refined byproduct of the sugar industry as molasses [4, 5].

The ability of *Escherichia coli*, an industrial biotechnology workhorse [6–8], to utilize sucrose as a sole carbon source depends on the specific *E. coli* strain used [9]. *E. coli* W is the only well-known *E. coli* strain generally regarded as safe that can utilize sucrose as a carbon source and can grow robustly on it when compared to other carbon sources, such as glucose [10]. The genetic basis and molecular control of sucrose metabolism in *E. coli* W (as well as *E. coli* EC3132) have been characterized [11, 12] and this knowledge is the basis for constructing sucrose utilizing *E. coli* strains for the current study. Engineering non-native sucrose utilization into additional *E. coli* strains has been investigated, but in all cases, the generated strains were characterized by slow growth rates on sucrose as compared to glucose. Such low growth rates would limit productivity in industrial processes, a key component for a viable bioprocess [13, 14]. Most of the previous approaches to engineer *E. coli* to use sucrose as a carbon source were hampered by slow growth rates and phenotypic instability due to unstable plasmid systems that may require antibiotic addition for stability [15] or due to a high burden exerted on the cell by high copy number plasmids [11, 16–18]. For example, the K-12 strains generated by Tsunekawa et al. using chromosomal integration grew with very slow rates on sucrose and random mutagenesis was required to improve the growth rate modestly [19]. Another approach examined was to chromosomally integrate sucrose utilization genes from *E. coli* W into an *E. coli* K-12 strain [20]. The K-12 strain generated was able to grow on sucrose, but the growth rate was 30% lower than that on glucose. Thus, there is still a need to efficiently engineer sucrose metabolism in multiple *E. coli* strains, each of which have their own strain-specific advantages for host selection for viable bioprocesses [6]. Such host selection factors to consider include product tolerance, phage resistance, the native metabolic flux distribution either towards a native or heterologous production pathway, transformation efficiency, convenience to perform metabolic changes, and ease to scale-up [21].

**Fig. 1:**
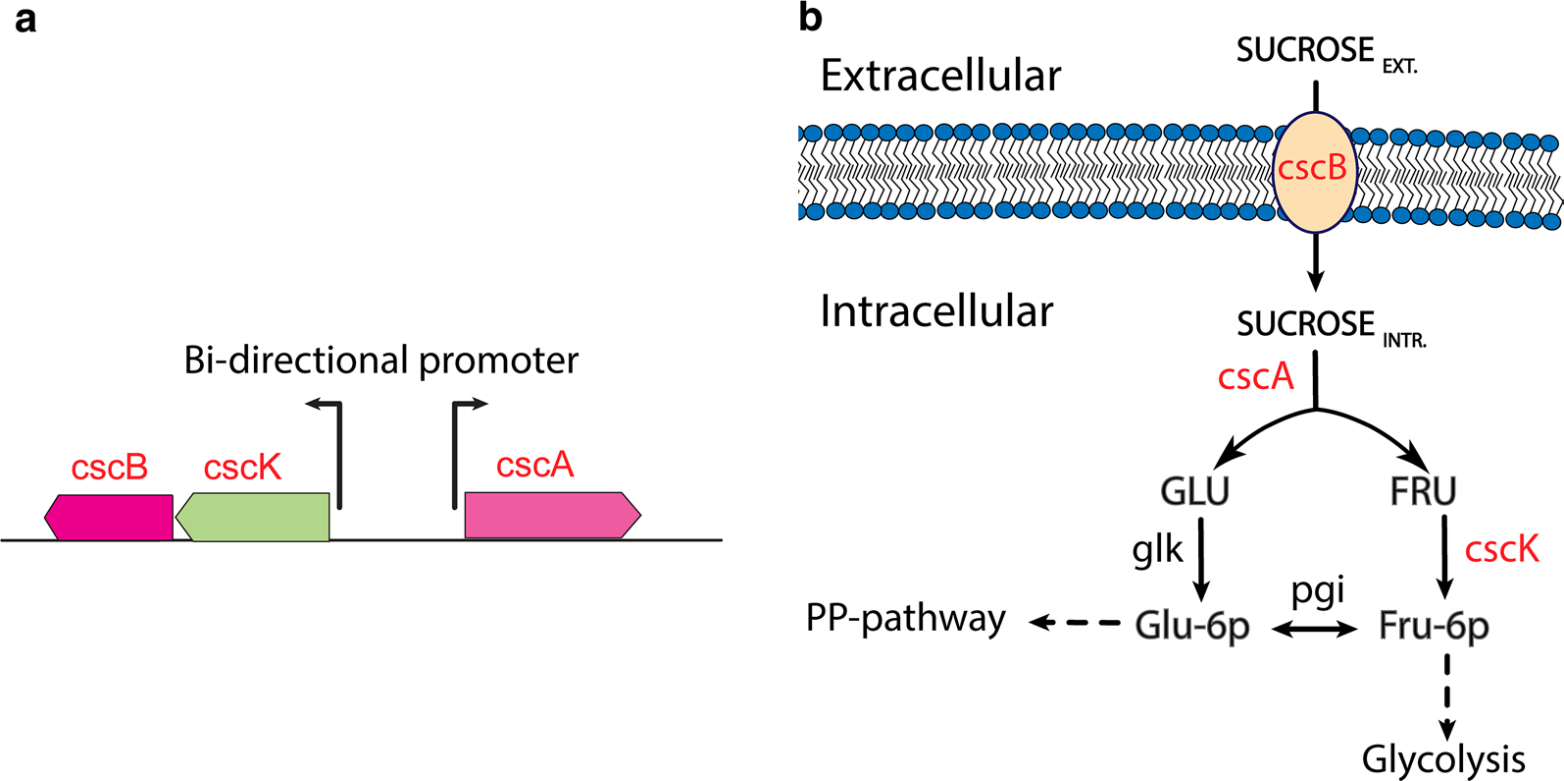
Schematic presentation of sucrose utilization mechanism with gene cassette integrated into *E. coli* K-12 MG1655 and route of sucrose entering into the central carbon metabolism. **a** The sucrose integrated cassette *csc* consists of *cscA*, *cscK*, and *cscB*. CscBK and *cscA* expression is derived by a bi-directional promoter. **b** Sucrose utilization starts with uptake of sucrose across the cell membrane by *cscB* (sucrose permease). Inside the cell sucrose is split into glucose and fructose by *cscA* (invertase). Glucose and fructose are then phosphorylated by glucokinase (glk) and fructokinase (*cscK*) respectively. Glucose-6-phosphate can be converted to fructose-6-phosophate by the isomerase (pgi) or enter directly into the pentose phosphate pathway (PP-pathway) or Entner-Doudoroff pathway (ED-pathway); fructose-6-phosphate enters the glycolysis directly

In order to demonstrate the ability to generate additional *E. coli* strains that can efficiently consume sucrose, genetic constructs were designed based on the chromosomally encoded sucrose catabolism operon, *csc*, from *E. coli* W and then subjected to adaptive laboratory evolution (ALE). The *csc* gene cluster containing the *cscB* gene that encodes sucrose permease, *cscK* that encodes a fructokinases, and *cscA* that encodes sucrose-6-phosphate hydrolase (invertase), was integrated into the *E. coli* K-12 MG1655 chromosome [11] (Fig. 1). The *cscR* gene that encodes for a *csc*-specific repressor and negatively controls the expression of the *csc* regulon was not integrated to allow constitutive expression. The engineered strain was then optimized using ALE to generate multiple evolved *E. coli* strains able to metabolize sucrose with fast growth comparable to that on glucose. Additionally, the approach to analyze multiple independent populations and multiple independent isolates from each population made it possible to effectively reveal key causal mutations by comparing the independent lineages and focusing on instances of parallel evolution. This approach also likely identified a broader landscape of mutations (e.g., multiple alleles of the same gene) as compared to sequencing a single lineage and reverse engineering all of the mutations to find causality. The ALE approach used in the current work used an automated platform with several parallel replicates as previously described [22, 23]. Overall, cells were repeatedly grown in a batch aerobic cultivation mode with passage in the exponential phase, which applies selection pressure for rapid growth rate. At the end of the evolution, populations and clones from the ALE derived endpoints were characterized in terms of their genome sequence, growth rate, carbon uptake rate, and growth yield. Furthermore, the evolved strains were characterized on sugarcane juice as a substrate demonstrating their ability to use this renewable feedstock efficiently.

## Methods and materials

### Media and reagents

#### M9 minimal medium

M9 sucrose medium contained 20 g/L sucrose unless stated otherwise, 1× M9 salts, 2 mM MgSO_4_, 100 μM CaCl_2_ and 1× trace elements and Wolfe’s vitamin solution. M9 salts, trace elements and vitamins were prepared in concentrated stocks. Stock M9 salts solution consisted of 10 × 68 g/L Na_2_HPO_4_ anhydrous, 30 g/L KH_2_PO_4_, 5 g/L NaCl, and 10 g/L NH_4_Cl dissolved in Milli-Q filtered water. M9 trace elements stock was a 2000x solution with composition of 3.0 g/L FeSO_4_·7H_2_O, 4.5 g/L ZnSO_4_·7H_2_O, 0.3 g/L CoCl_2_·6H_2_O, 0.4 g/L Na_2_MoO_4_·2H_2_O, 4.5 g/L CaCl_2_·H_2_O, 0.2 g/L CuSO_4_·2H_2_O, 1.0 g/L H_3_BO_3_, 15 g/L disodium ethylene-diamine-tetra-acetate, 0.1 g/L KI, 0.7 g/L MnCl_2_·4H_2_O and concentrated HCL dissolved in Milli-Q filtered water. Vitamin mix and trace elements concentration was [1×] in the M9 medium.

#### Sugarcane juice medium

Raw sugarcane juice was collected from a sugar cane mill (Enterprise Factory, Patoutville, Louisiana, USA) and transported to the University of Illinois Energy Farm where it was stored at − 80 °C until required for further use. Sugarcane juice (SCJ) for the cultivation medium was prepared by centrifugation in order to remove solid particles (rpm ≥ 10,000×*g* for 20 min at 4 °C) then filter sterilized with 0.2 µm pore size filter. Later, Sugarcane juice (SCJ) medium was prepared by mixing clarified sugarcane juice with M9 salts (final concentration 1×), 2 mM MgSO_4_, 100 μM CaCl_2_ and 1× trace elements and Wolfe’s vitamin solution. HPLC analysis of the clarified sugar cane juice showed the following sugar composition (g/L): sucrose 231.8, glucose 3.5; fructose 3.0.

### Strains constructions with cscBKA cassette using homologous recombination

General recombinant DNA techniques were applied according to standard protocols for one-step cloning and chromosomal integration of DNA (pOSIP), Additional file 2: Text file S1 [24, 25]. PCR products were purified using plasmid miniprep kit, QIAprep Spin Miniprep Kit. Here, the integration site of *csc* DNA sequence was into *E. coli* 186 attB site using pOSIP-KO integration module. The integration site of DNA sequence into bacterial chromosomes was selected based on the available integration sites. Csc gene cluster (*cscK*, *cscB*, *cscA*) (Fig. 1a) was amplified from donor strain, *E. coli* W using primers; Forward primer 5′-ATGCATCUGGGATATAGAGCTATCGACAACAACCG-3′ and Reverse primer 5′-AGAGGGCUTTATGTTAACCCAGTAGCCAGAGTGCTC-3′. *E. coli* K-12 construct with *csc* cassette integrated was abbreviated to MGcscBKA. MGcscBKA derivative with a variant SNP in *cscB* Q353H was abbreviated MGcscBKAp [16].

### Adaptive laboratory evolution of developing improved fitness in start strains

Three biological replicates from each of *E. coli* strains; two genetic constructs of K-12 MG1655 with csc gene cassette MGcscBKA and MGcscBKAp and *E. coli* W strain, were grown overnight in M9 minimal media with 20 g/L sucrose. On the next day 150 µL of the overnight culture was passed into a new fresh 30 mL tube filled with a total working volume of 15 mL M9 medium (i.e., a 1:100 ratio) with 20 g/L sucrose as the sole carbon source. Bacterial cells were serially passaged during exponential growth phase for approximately 40 days using an automated liquid-handler platform as described by Lacroix et al. [22]. The liquid-handler platform was utilized to automate executing multiple evolution experiments at the same time. It has the capacity to aspirate cell cultures when passing from a mature culture tube to a freshly filled tube based on a pre-defined media recipe in a sterile strictly-controlled platform, in an effort to keep the cells growing and passing at the exponential phase of growth. The commonly encountered exponential phase was from the time of the inoculation to an approximate optical density of 600 nm (OD_600nm_) of 1.3–2.0 in order to keep the cells under a constant selection pressure. Cells were cultured in a heat block at 37 °C with magnetic stir bar for full aeration at 1200 rpm. Periodically, OD_600nm_ was measured at a time determined by a predictive script and once OD_600nm_ reached approximately 1.3 using Sunrise plate reader (Tecan, Männedorf, Switzerland), 150 µL was passed into a tube with a 15 mL working volume of fresh media. The common conversion factor between the plate reader used and the Benchtop spectrophotometer is 4.2. This process was repeated until a significant increase in fitness was achieved. Periodically, glycerol cryogenic stocks were prepared and stored at − 80 °C for any culture restarting. Endpoint population samples were streaked on agar plates to select single clones for whole genome resequencing.

### Genome sequencing and mutation calling of the ALE derived strains

Overall, there were six endpoint populations for each of K-12 MG1655 constructs and 3 endpoints populations from *E. coli* W were selected along with clones derived from for whole genome re-sequenced in order to reveal their underlying genotypes. Genomic DNA was extracted from overnight cultures at the stationary phase of growth using PureLink^®^ Genomic DNA extraction kits (Invitrogen, CA). The quality of extracted DNA was assessed by using a Nanodrop spectrophotometer. Concentration of the extracted DNA was quantified using Qubit ds-DNA high sensitivity assay. Paired-end re-sequencing libraries were generated using a 300 cycle (150 bp × 2) kit from Illumina (San Diego, CA) with loading concentration of 1.2 pico-Molar on Illumina Nextseq sequencer (Model 550). Mutation finding was performed using a pipeline as described in Phaneuf et al. [26] based on *Breseq* version 0.30.1 [27] to map sequenced reads to the reference strain (NCBI accession number NC_000913.3, K-12 MG1655 and NC_017664, W). The average coverage for each of the resequenced samples was over 25x. For population samples sequenced, mutations were reported if they were over 20% frequency unless they were found in a clone isolated from a given population sample. In this case, the frequency of a clone mutation was reported, if found, in the population (see Additional file 1: Data file S1).

### Validation of mutations causality in *rpoB, rpoC* and *pyrE*-*rph* genes

Identified causal mutations found in the current study (see mutation analysis results) and the control reference ALE experiment by LaCroix et al. [22] on glucose (GLUALE) were used to check causality in the identified genes or genetic regions on either sucrose and glucose minimal media. Briefly, the identified key single point mutations (SNPs) in open reading frames (ORF) mainly for *rpoB*, *rpoC* genes and the unique deletion in the intergenic region between *pyrE*/*rph* were used to check the causality of mutations in these specified genetic regions with sucrose for growth increase from the two experiments. Accordingly, selected isolates from ALE derived clones on sucrose which harbor any single mutation or double mutations in these genes were selected as well. Fitnesses increase comparison relative to the starting strain were examined to investigate the effect/essentiality of the key causal mutations for fast growth on either sucrose or glucose as a sole carbon source. The same recombinant DNA technique used to generate MGcscBKA constructs with the *csc* regulon was applied here to generate GLUALE constructs with a sucrose utilization cassette (GLUALE_csc constructs).

### Extracellular metabolites and physiological properties

Cultures of the re-sequenced clones were inoculated from stationary phase overnight cultures into media M9 containing sucrose under the same conditions as the ALE experiment. Samples were aliquoted over the growth curve to measure optical density OD_600nm_ and collect extracellular metabolites. Extracellular metabolites were collected as supernatant from each growing culture using 0.2 µm filter to remove the cells. Supernatants were collected and saved at − 20 °C for subsequent chromatographic analyses. Concentration of sugars (glucose, fructose and sucrose) beside other organic acids were analyzed using high performance liquid chromatography (HPLC) column (UltiMate 3000, Thermo-Fischer Scientific, Waltham, Massachusetts, USA). The metabolites were separated using an Aminex HPX-87H ion exclusion column (Bio-Rad, Hercules, California, USA) and were isocratically eluted at 30 °C, with a flow rate of 0.6 mL/min, using a 5 mM sulfuric acid solution as mobile phase. The refractive index (RI) detector was selected for detection. Sample concentrations were quantified by comparing to a standard curve of known concentrations. Substrate uptake and metabolites excretion rates were calculated from multiplying the growth rate and the slope of a linear regression of gram dry cell weigh (gCDW) versus the substrate or products concentration. ﻿The biomass yield at the steady state (Y_X/S_ss_) was calculated as the quotient of the growth rate and the sucrose uptake rates during the exponential growth phase.

## Results

### Evolution of multiple *E. coli* strains to grow rapidly on sucrose minimal media

ALE was utilized to generate strains with improved fitness utilizing sucrose as a sole carbon source. Three different *E. coli* starting strains were used; two engineered K-12 MG1655 strains with sucrose utilization enabling csc constructs and wild-type *E. coli* W. The two constructs inserted into the K-12 MG1655 host genome differed by a SNP mutation in the *cscB* gene, resulting in the residue change Q353H, and were labeled as MGcscBKA and MGcscBKAp (see Additional file 2: Text S1 for a detailed description). The Q353H derivative of *cscB* has been demonstrated to display an increased sucrose uptake rate in *E. coli* EC3132 [16], thus it was reasoned that it may have an impact when heterologously expressed in K-12 MG1655.

**Fig. 2:**
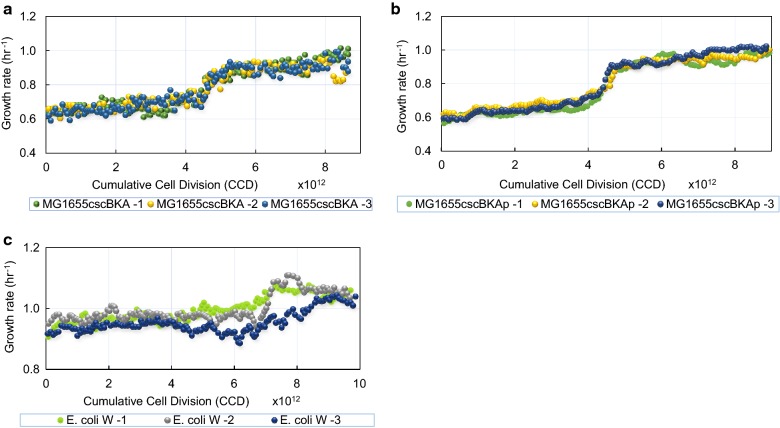
Plots of population fitness (i.e., growth rate) trajectories during the ALE experiments on sucrose. Three biological replicates of each of the *E. coli* strains: **a** K-12 MG1655cscBKA, **b** K-12 MG1655cscBKAp, and **c** W were evolved on 20 g/L sucrose minimal medium. Shown are the growth rates versus cumulative cell divisions (CCD) for the three biological replicates for each strain

Three independent populations of each starting strain were evolved in batch and in parallel under the strict selection pressure of continuous exponential growth for approximately 40 days (Table 1, Fig. 2a–c). Termination of the ALE experiment was determined based on two key parameters [28]; the delta change in growth rate (Δμ) and the passage size. Therefore, the experiments were terminated when the parallel replicates showed a small to no delta change in the growth rate similar to the control ALE on glucose [22], with the passage volume equal to 1%. Each of the replicate evolutions underwent between approximately 8.65 × 10^12^ to 9.87 × 10^12^ cumulative cell divisions (CCD, Table 1). The use of CCD has been demonstrated as a useful timescale for ALE experiments as a time coordinate as it accounts for variability due to a varying number of cells passed serially from one flask to the next [29].

**Table 1:** Properties of the ALE experiments end point populations

ALE experiment	Strain (population)	Total CCD x10^12^	Ratio of fitness increase to start strain
K-12 MGcscBKA	1	8.65	1.72
	2	8.75	1.46
	3	8.94	1.61
K-12 MGcscBKAp	1	9.02	1.48
	2	9.02	1.47
	3	8.85	1.48
*E. coli* W	1	9.71	1.29
	2	9.78	1.31
	3	9.87	1.22

Ratios of fitness increase were determined from growth rates that were calculated based on the last three flasks during exponential growth. CCD, cumulative cell divisions

The observed growth rate trajectories during the ALE experiments for each of the evolved populations are shown in Fig. 2 for the MGcscBAK, MGcscBKAp, and *E. coli* W strains. Each population displayed an increase in growth rate over the starting strain (Table 1). The growth rate increases were 1.49 ± 0.098-, 1.75 ± < 0.01-, 1.25 ± 0.036-fold faster than the starting strains for the MGcscBKA, MGcscBKAp and *E. coli* W strains, respectively (standard deviation, n = 3). There was one observed fitness jump (i.e., increase in growth rate) for all the populations across all independent ALE replicates. The two similar K-12 MGcscBKA and MGcscBKAp strains evolved to relatively similar growth rates, with similar dynamics along the course of the ALE, whereas the fold increase in fitness for *E. coli* W was small given its initial faster wild-type growth rate.

### Physiological characterization of evolved clones

**Fig. 3:**
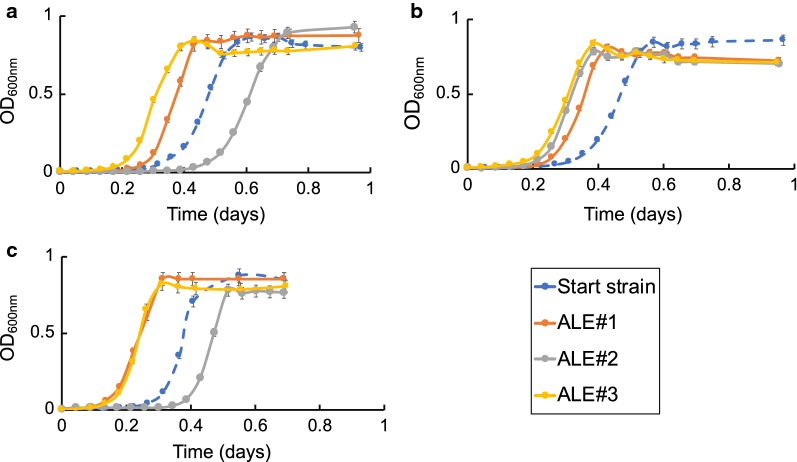
Growth profiles of ALE clones isolated from the end populations of the evolution experiments versus starting strains on M9 minimal medium with 20 g/L sucrose. Error bars represent standard deviation (n = 3). MG1655 construct with *csc* (MGcscBKA) growth profile is represented on **a**, MGcscBKA derivatives with *cscB* SNP (MGcscBKAp) mutation on **b** and *E. coli* W strain on **c**. Optical density measurement at OD_600_ were measured on a plate reader and ﻿the common ratio between the plate reader OD_600_ and a benchtop spectrophotometer with a 1 cm path length is 4.2

Clones from the endpoint populations of each of the nine replicate ALEs were isolated and characterized in terms of growth rate and compared to the starting strains to understand the enhanced evolved phenotype. One clone isolated from each replicate endpoint population was analyzed, three clones for each of the three replicates of MGcscBAK, MGcscBKA, and *E. coli* W. The increases in growth rates on sucrose M9 were between 1.17 and 1.57-fold for MGcscBKA clones, 1.25–1.38-fold for MGcscBKAp, and 1.06–1.22-fold for *E. coli* W, Table 2. Surprisingly, there was one isolated clone that had the same fitness, i.e. growth rate, as the starting strain from MGcscBKA (the ‘#2’ clone) (Fig. 3). However, given that there were two additional clones from this starting strain from the additional two independent replicates with a significant improvement in fitness, additional clones were not examined.

**Table 2:** Physiological data for the evolved isolates on sucrose M9 medium and sugarcane juice M9 minimal medium (SCJ medium)

Clone/construct	Clone ID	M9 Sucrose medium	SCJ medium					
		Growth rate, μ (h^−1^) on M9 2% sucrose	Growth rate, μ (h^−1^)	Final density (gCDW/L)	Sucrose uptake rate (mmol gCDW^−1^ h^−1^)	Acetate production rate (mmol gDW^−1^ h^−1^)	Biomass yield, Y_X/S_, (gCDW g of sucrose^−1^)	Uptake rate fold increase vs start strain
K-12 MGcscBKA	Starting strain	0.54 ± 0.01	0.68 ± 0.03	1.47 ± 0.04	7.56 ± 0.96	4.20 ± 0.20	0.28 ± 0.01	1.00
	1	0.85 ± 0.01	0.88 ± 0.02	1.91 ± 0.03	8.71 ± 0.2	4.73 ± 0.11	0.30 ± 0.01	1.15
	2	0.63 ± 0.01	0.68 ± 0.02	1.52 ± 0.04	6.54 ± 0.23	5.67 ± 0.54	0.30 ± 0.01	0.86
	3	0.83 ± 0.01	0.86 ± 0.02	1.68 ± .0.03	7.84 ± 0.18	5.42 ± 0.13	0.32 ± 0.01	1.04
K-12 MGcscBKAp	Starting strain	0.64 ± 0.01	0.73 ± 0.01	1.48 ± 0.02	7.01 ± 1.9	4.99 ± 0.25	0.30 ± 0.01	
	1	0.86 ± 0.01	0.91 ± 0.01	1.38 ± 0.02	9.89 ± 0.19	6.83 ± 0.01	0.27 ± 0.01	1.41
	2	0.88 ± 0.01	0.88 ± 0.01	1.44 ± 0.02	9.50 ± 0.01	8.27 ± 0.01	0.27 ± 0.01	1.36
	3	0.80 ± 0.01	0.82 ± 0.01	1.51 ± 0.01	8.98 ± 0.11	7.30 ± 0.09	0.27 ± 0.01	1.28
*E. coli* W	Starting strain	0.90 ± 0.02	0.97 ± 001	1.81 ± 0.05	9.88 ± 0.35	6.60 ± 0.49	0.29 ± .01	
	1	1.10 ± 0.01	1.06 ± 0.05	1.68 ± 0.06	10.5 ± 0.47	15.90 ± 0.68	0.29 ± 0.012	1.06
	2	0.95 ± 0.01	0.96 ± 0.02	1.74 ± 0.05	14.10 ± 1.0	6.50 ± 0.51	0.20 ± 0.02	1.43
	3	0.95 ± 0.01	1.08 ± 0.02	1.92 ± 0.01	12.51 ± 1.1	7.40 ± 0.5	0.25 ± 0.03	1.27

The physiological properties of each of the clones isolated from the independent endpoint ALE experiments were compared to examine whether there were any improved phenotypic outcomes across the different experiments

**Fig. 4:**
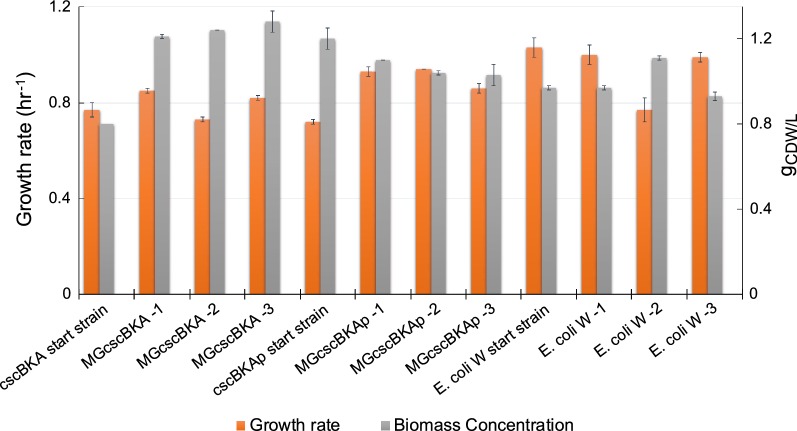
Characterization of the isolated sucrose-evolved clones on 2 g/L glucose M9 minimal medium. Shown is a stacked histogram of growth rate (h^−1^, orange) of the selected clones and their corresponding final cellular density (gCDW/L, grey). The plot shows that mutations acquired during the ALE experiment on sucrose were also largely beneficial, to a similar extent, on glucose as a sole carbon source. Error bars represent standard deviation of biological replicates (n = 3). Data is shown for both the starting strains and evolved clones for the three different sets of evolved strains

Furthermore, growth profiling of all of the sucrose-evolved endpoint clones was performed in glucose M9 media. In agreement with the observed sucrose M9 phenotypes, all clones demonstrated an increased growth rate over the start strains without any significant changes to the final cellular density (gCDW/L) (Fig. 4). One exception to this was *E. coli* W replicate #2 which exhibited a slightly lower growth rate than the starting strain. The observation that the vast majority of the sucrose-evolved strains also displayed a fast growth phenotype on glucose is an indication that most mutations acquired during the sucrose ALE experiment were beneficial for growth on both sugars. Such a phenotype is advantageous for a platform strain as it could be used in multiple media conditions. All isolated clones were then sequenced to examine their genetic basis along with population sequencing to better understand the genotypes responsible for the observed increases in population fitness from all of the independent ALE experiments.

### Whole genome sequencing and mutation analysis

Whole genome sequencing was used to determine the genetic basis of the improved fitness phenotypes for the evolved *E. coli* strains on sucrose minimal medium. The nine isolated endpoint clones were sequenced along with the corresponding populations from which they were isolated. Overall, there were nine genes or genetic regions identified from the K-12 strains found in clones and/or populations (frequency cutoff ≥ 0.20) that were mutated either once or several times across the parallel independent replicates. This number was five for *E. coli* W. Additionally, there was a range of 1-3 unique mutations found in all of the clones sequenced, with most clones possessing one mutation. When looking at the population data, there was a range of 0-3 mutations detected in each sample with a frequency cutoff ≥ 0.20, indicating different levels of clonal interference in different replicates. Key mutations were identified by comparing all clones and population samples sequenced and identifying open reading frames (i.e., genes), or intragenic regions that had multiple unique mutations or were mutated across independent experiments (Table 3). Interestingly, there were no shared key mutations found between the K-12 and *E. coli* W experiments. Full mutation lists for each sequenced sample are given in Additional file 1: Data file S1.

**Table 3:** Key mutations found after evolution of *E. coli* on sucrose

Strain	Gene or genetic region	Mutation	Mutation type	Function	Number of independent occurrences	Sample ID (frequency of mutation in population samples)
K-12 starting strains MGcscBKA and MGcscBKAp	*pyrE/rph*	Δ82 bp intragenic	DEL	Orotate phosphoribosyltransferase/ribonuclease PH	2	MGcscBKA A1 F136 I0 (43%), MGcscBKA A1 F136 I1, MGcscBKA A3 F140 I0 (25%)
		T→G intergenic (− 47/+ 19)	SNP		1	MGcscBKAp A3 F139 I0 (71%)
	*rpoB*	S621F (TCC→TTC)	SNP	RNA polymerase subunit β	1	MGcscBKA A1 F136 I0 (48%), MGcscBKA A1 F136 I1
		Q618L (CAG→CTG)	SNP		1	MGcscBKA A2 F134 I0 (4%)
	*rpoC*	T1045P (ACC→CCC)	SNP	RNA polymerase subunit β’	2	MGcscBKA A3 F140 I0 R1 (20%), MGcscBKA A3 F140 I1 R1, MGcscBKAp A3 F139 I0 (83%)
		R1075C (CGT→TGT)	SNP		2	MGcscBKAp A1 F138 I0 (93%), MGcscBKAp A1 F138 I1, MGcscBKAp A2 F140 I0 (100%), MGcscBKAp A2 F140 I1
		L770R (CTC→CGC)	SNP		1	MGcscBKA A3 F140 I0 (76%)
		R978C (CGT→TGT)	SNP		1	MGcscBKAp A3 F139 I0 (9.7%), MGcscBKAp A3 F139 I1
		Q665K (CAG→AAG)	SNP		1	MGcscBKA A2 F134 I0 (6%)
		R1174P (CGT→CCT)	SNP		1	MGcscBKA A2 F134 I0 (18%)
*E. coli* W	*cscR*	Δ1403 bp [cscR, dsdX, dsdA]	DEL	Sucrose operon repressor (Csc operon regulatory protein), permease DsdX, d-serine dehydratase (EC:4.3.1.18)	1	*E. coli* W A1 F155 I1
		Δ84 bp	DEL	Sucrose operon repressor (Csc operon regulatory protein)	1	*E. coli* W A3 F156 I1
		Δ10 bp	DEL		1	*E. coli* W A1 F155 I0 (74%)
		Δ21 bp	DEL	Sucrose operon repressor (Csc operon regulatory protein)	1	*E. coli* W A3 F156 I0 (46%)
	mrdB	S31R (AGC→AGG)	SNP	Rod shape-determining protein RodA	1	*E. coli* W A1 F155 I0 (77%)
		S37R (AGC→AGA)	SNP		1	*E. coli* W A2 F158 I0 (81)
		E270Q (GAA→CAA)	SNP		1	*E. coli* W A2 F158 I1
	mrdA	W434C (TGG→TGC)	SNP	Penicillin-binding protein 2	1	*E. coli* W A1 F155 I1

The sample ID has a unique identifier—(A) refers to the independent replicate, (F) refers to the flask number, and (I) to the type of the isolate: population (0) or clone (1)

The most predominantly mutated genes or intergenic regions identified across multiple independent replicates in the evolved K-12 strains were related to the RNA polymerase subunits *rpoB* and *rpoC*, as well as orotate phosphoribosyltransferase, *pyrE*. The first key mutations were found in the β (beta) and β’ (beta prime) subunit of RNA polymerase encoded in *rpoB* and *rpoC,* respectively [30, 31]. The number of unique mutations for each of *rpoB* and *rpoC* found across parallel populations and clones was 2 and 6 unique SNPs, respectively. Both *rpoB* mutations occurred in one region between amino acids residues (AAR) 618 and 621, whereas the *rpoC* mutations occurred closer to the carboxyl terminus (AAR total 1342) of the beta prime subunit at AAR between 665 and 1174, Table 3. There was one co-occurrence of a *rpoC* mutation, T1045P (ACC→CCC), which was observed in experiments starting from both K-12 strains. Interestingly, mutations in *rpoB* and *rpoC* genes have previously been found repeatedly across all different K-12 ALE experiments, indicating a very high level of parallel evolution [22, 32, 33]. It is also interesting to note that no clone contained mutations in both genes, but there were often multiple mutations in these genes found in population samples (never adding up to more than approximately 1 in mutation frequency). Specific mutations in these two subunits of the RNA polymerase, i.e. the beta and the beta prime, were found to carry beneficial growth advantages when *E. coli* grows on minimal medium with a range of different carbon sources [22, 32–36]. The second key mutated region observed in K-12 strains were related to *pyrE* expression. K-12 strains are known to possess a frame shift in *rph* which leads to pyrimidine starvation on minimal media and can be alleviated by mutations [37]. Similar mutations were reported in different ALE studies on different carbon sources [32, 34]. Mutations in this genetic region were found to improve fitness on minimal media with an increase in growth rate of 17% over the starting strain on glucose [22].

Strain-specific adaptive mutations in *E. coli* W clones were predominantly affecting a metabolic regulation pathway targeting the *csc* operon and cell wall biosynthesis through rod shape determining proteins. Many of the mutations found related to the *csc* operon were in the *cscR* gene, which encodes a transcriptional repressor regulator for *csc* operon (*cscB*, *cscA*, *cscK* genes) in low concentrations of sucrose [11, 16]. A cscR mutation was observed in two endpoint clones (out of three) and a total of four mutations relating to this gene were found overall when considering population sequencing. The clonal cscR mutations were an intragenic in-frame Δ84 bp deletion and an Δ1403 bp deletion which also included the *dsdX* and *dsdA* genes located next to *cscR* on the chromosome. Both mutations are likely a disruption of the cscR gene with potentially a loss of function. The two genes *dsdX* and *dsdA* are pseudogenes coding for a d-serine transporter and d-serine ammonia-lyase, respectively, as part of serine degradation pathway [38, 39]. Deletions of *cscR* were previously demonstrated to improve growth and yield for chemical bioproduction [12, 40]. Another pair of genes that were mutated several times along parallel experiments is *mrdB* (three times) and *m*rdA (one time). MrdB is annotated as rod shape-determining protein RodA [41, 42] and the related *mrdA* is annotated as penicillin-binding protein 2 [43]. All of the mutations found in the *mrdB* and *mrdA* genes were SNPs changing the properties of the protein through single amino acid changes (see Table 3). The impact of each SNP on the protein functionality or activity was not immediately clear based on the known structural data for these genes. Mutations in the *mrdA* and *mrdB* genes were reported in a previous temperature tolerance ALE experiment [44]. Mutations in *mrd* loci encoding the elongasome such as *mrdA* and *mrdB* have been shown to increase the levels of the growth-rate-regulating molecule (p)ppGpp, which potentially can lead to carbon metabolism modulation [45]. Additionally, a causal mutation in the cell shape determining gene *mrdA* was identified in ALE for developing osmotolerant *E. coli* strains [46].

## Validation of mutational causality by reverse engineering

**Fig. 5:**
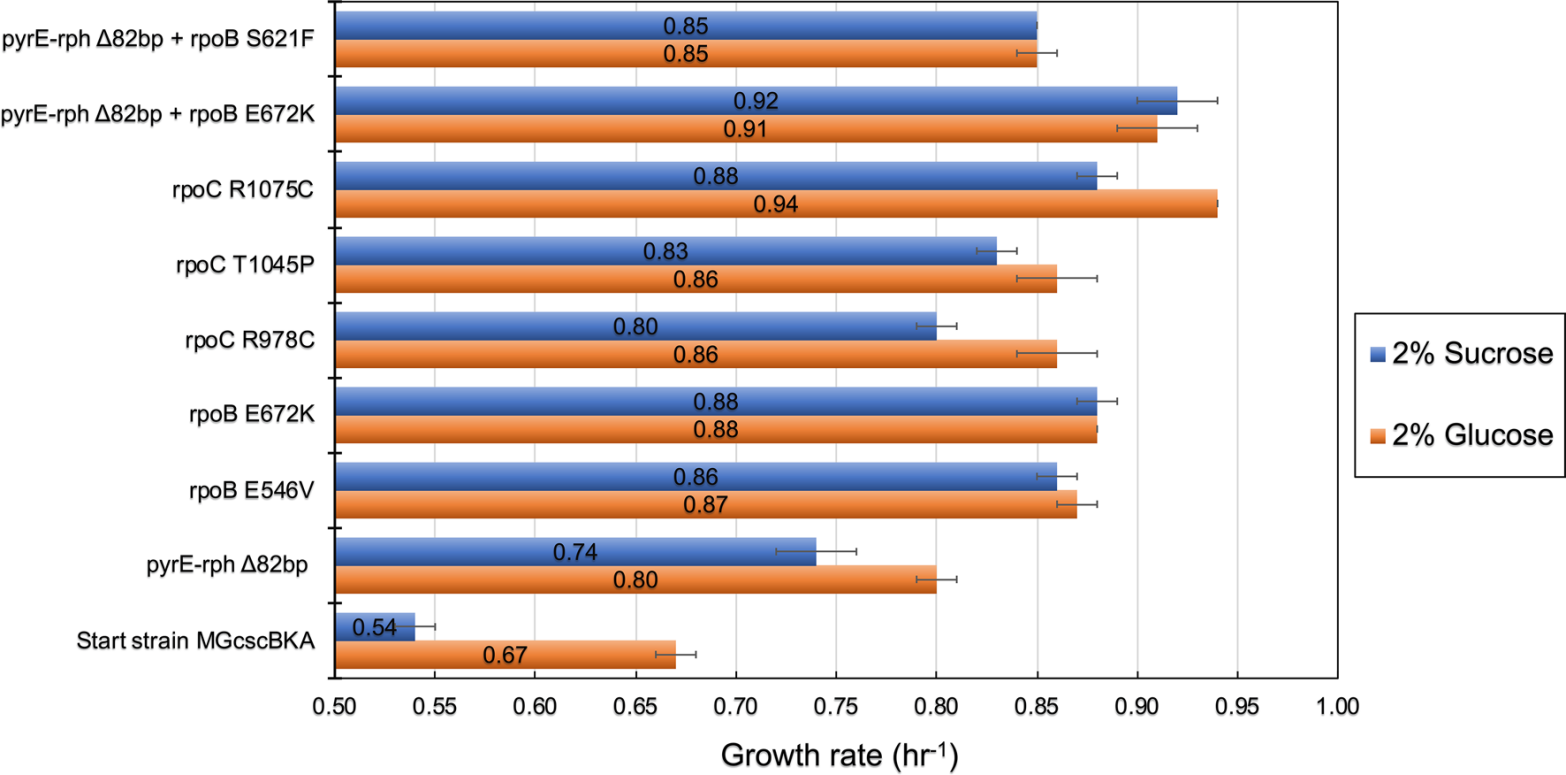
Causal mutation analysis for fitness improvement on sucrose and glucose for K-12 strains. Shown is a bar graph representing growth rate of identified causal mutations from the current study (ALE on sucrose) and LaCroix et al. [22] (ALE on glucose). The error bars are calculated from two biological replicates (n = 2). All *rpoC* mutations originated from this study, as well as the *rpoB* S621F with the *pyrE*-*rph* deletion mutation (i.e., they were not reverse engineered). The remaining mutations were from glucose ALE control experiment (GLUALE) with chromosomally integrated csc gene cassette as described in the methods

To examine the causality of key mutations identified in this study, growth screens were performed for relevant single and double mutant strains of K-12 MG1655. Such mutant strains (see methods, validation of mutation causality) were either isolated directly from the current study (i.e., endpoint clones containing the csc construct and mutations) or generated from previously constructed mutant strains [22] which were subsequently engineered to also contain the *csc* gene cassette (see Table 4). Growth screens were performed side by side on M9 medium supplemented with either sucrose or glucose (20 g/L). Genes investigated for potential causality for improved fitness, i.e., higher growth rate, were *rpoC*, *rpoB* and the intergenic deletion ∆82 bp between *pyrE*/*rph*. To put these results in context, ALE-derived mutations in these exact genes were validated previously to confer fitness advantages in various substrate environments, and in particular when grown on glucose [22, 32, 33]. Figure 5 provides comparative fitness levels for each adaptive mutation on either sucrose or glucose.

**Table 4:** Source of key mutations validated for causality in isolated strains from the current study or constructed previously from Lacroix et al. [22]

Genetic region	Mutation	Source
K-12 MG1655	N/A	N/A
*pyrE*-*rph*	Δ82 bp deletion	LaCroix et al. [22]
*rpoB*	E672K (GAA→AAA)	LaCroix et al. [22]
*rpoC*	R1075C (CGT→TGT)	MGcscBKAp A1 F138 I1 MGcscBKAp A2 F140 I1
	R978C (CGT→TGT)	MGcscBKAp A3 F139 I1
	T1045P (ACC→CCC)	MGcscBKA A3 F140 I1 with additional mutations of (*tdcG*, *baeS*)
*pyrE*-*rph* + *rpoB*	Δ82 bp + E672K (GAA→AAA)	LaCroix et al. [22]
*pyrE*-*rph* + *rpoB*	Δ82 bp + S621F (TCC→TTC)	MGcscBKA A1 F136 I1

The comparative analysis of reverse engineered strains showed that mutated genes identified in this ALE study conferred a fitness advantage over the starting strain and some were additive in their impact on growth rate. Interestingly, the fitness advantage of all tested mutations over the starting strain was observed when grown on M9 with either sucrose or glucose as carbon sources, and the fitness level increases for a given mutated strain were roughly similar when grown on sucrose or glucose. For the mutations examined, the RNA polymerase mutations have a higher increase in growth rate over the causal mutation affecting nucleotide biosynthesis (*pyrE*). Furthermore, analysis showed that when multiple mutations were present in a strain, such double mutant strains conferred a higher fitness advantage over single mutants with the identical mutations. Specifically, a strain with a mutation in the intragenic region between *pyrE*/*rph* genes and in *rpoB* (E672K) had higher fitness than strains with either single mutations. This pattern of co-occurrence was observed when evolving K-12 MG1655 on either glucose [22] or sucrose (endpoint isolate derived from MGcscBKA ALE#1 population from the current study).

### Benchmarking of evolved strains performance on sugarcane juice

To further evaluate the ALE-derived clones for sucrose utilization, each endpoint clone was characterized on a raw feedstock, sugarcane juice (SCJ) medium. A M9 medium base was utilized with SCJ to test both evolved and starting strains (Table 2 and Additional file 2: Figs. S1–S3). Raw SCJ mainly consists of three carbon sources; sucrose with the highest fraction, and glucose and fructose in small fractions [47]. The sucrose uptake rates (SURs) and acetate production rates (APRs) for all of the isolated clones were compared to each of the starting strains. Overall, there was an increase in the SUR for the K-12 MG1655 and *E. coli* W endpoint clones. The average increase in SUR was 1.10- ± 0.05- and 1.35 ± 0.05-fold increase for MGcscBKA and MGcscBKAp isolates, respectively, and a round 1.2 ± 0.2-fold increase for *E. coli* W isolates. Analysis of the MGcscBKAp starting strain showed a slightly lower SUR than that of the MGcscBKA starting strain. This finding was in contrast to the observation that such a mutation in *cscB* leads to a two-fold increase in sucrose uptake rate in a different *E. coli* strain [16]. However, the evolved MGcscBKAp clones demonstrated higher uptake rates than the MGcscBKA evolved clones (Table 2). Thus, it appears that there was an impact from this mutation at faster growth rates displayed by the evolved clones, but not the starting strains. However, reverse engineering of this mutation in the mutated endpoint strains of the MGcscBKA evolved clones would be needed to show this definitively.

As a general observation on the growth profiles of the evolved isolates, the initial amounts of glucose and fructose (approximately, 1–2 mM) were first depleted followed by the consumption of sucrose (Additional file 2: Figs. S1–S3). Interestingly, during the exponential growth phase, fructose and glucose accumulated in the culture broth in some replicates (e.g., *E. coli* W endpoint replicate #1, Additional file 2: Fig. S1) and was then consumed near the end of the aerobic cultivation. It was also observed that acetate was produced and accumulated during the course of the cultivation for all the tested clones except for the *E. coli* W clones (Additional file 2: Figs. S1–S3). In the evolved W clones, acetate accumulated up until approximately 10 h after inoculation, and then it was consumed as the sucrose, glucose, and fructose were depleted.

**Fig. 6:**
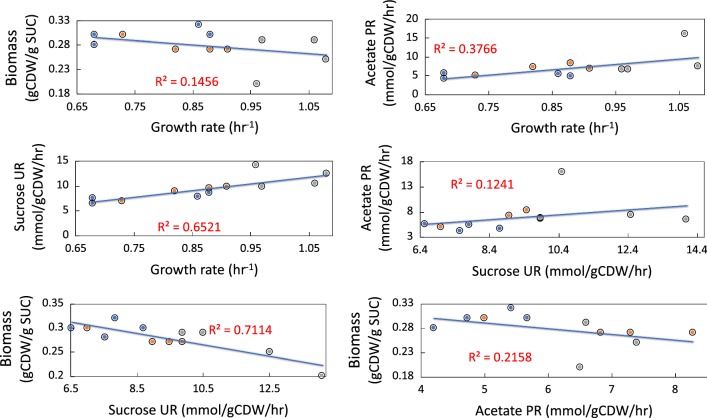
Pairwise comparisons of the phenotypic properties of the evolved clones on sugarcane juice M9 minimal medium (SCJ medium). Blue filled circles correspond to MGcscBKA clones, orange filled circles to MGcscBKAp clones and grey filled circles to *E. coli* W. Overall, sucrose uptake rate versus biomass yield and growth rate versus sucrose uptake rate were the highly correlated. Calculations can be seen in the methods section

The physiological profiling of selected endpoint isolate on SCJ were compared to examine whether there were any correlations or observed trends across the different evolved replicate experiments. Although the evolved clones isolated from the endpoints of the ALE experiments mostly showed a similar increase in fitness, the SUR and biomass yields varied more significantly (Table 2). However, the SURs and APRs were most often higher in endpoint clones compared to the starting strains (the exceptions being one clone, MGcscBKA isolate #2, in which SUR was not significant). There was a weak correlation (r^2^ = 0.12) between the increase observed in both SUR and APR, Fig. 6. Alternatively, there was a stronger correlation between the increase in SURs and APRs and the increased growth rate with correlations of (r^2^ = 0.65) and (r^2^ = 0.37), respectively. For the characterized clones, a trade-off between the SURs and biomass yield at the steady state (Y_X/S_) was observed where higher SURs led to lower biomass yields, i.e. an inverse correlation with an r^2^ = 0.71. The results implied that there were multiple mutational mechanisms through which a similar increase in the growth rate could be achieved (i.e., alternative optimal solution). The observed variations in SURs, APRs, and biomass yields further showed that under the selection pressure applied via ALE, the populations adapted through different trajectories in order to reach an apparent optimum metabolic state enabling a fast growth phenotype on sugarcane juice.

## Discussion

Platform strains that are able to exploit sucrose as abundant cheap carbon source from raw materials sources such as molasses and sugarcane juice can contribute to the increased economic and environmental sustainability of bio-based chemical production. Most of previous attempts to engineer a fast-growing K-12 strain on sucrose, to the same rate as growth on glucose, have not been successful. Thus, the scope of the current study was to generate a platform strains able to utilize sucrose as a carbon source and grow rapidly using adaptive laboratory evolution. Accordingly, the main findings from the current work are: (1) it was possible to effectively generate platform strains with elevated growth rates in a sucrose feedstock environment, which includes validation on raw material sugarcane juice. Moreover, the developed platform strains are suitable as fermentation chassis with an extended capability to also rapidly utilize glucose to the same level as strains evolved to grow solely on glucose [22]; (2) this study establishes a transferable sucrose utilization cassette for use in K-12 strains where sucrose utilization is not native; and (3) the identification of key reproducibly-occurring mutations using multiple parallel replicate ALE experiments. These mutations were validated using knock-in strains and can be used as cell engineering parts in additional strains of interest.

The ALE approach utilized in this work was successful in generating strains with improved fitness over their respective starting strains after approximately 40 days of continuous passaging in the exponential growth phase. The selected clones derived from the evolved populations for the K-12 strains exhibited increased carbon uptake rates for both sucrose and glucose, making them suitable for both carbon sources. Furthermore, the approach utilized in this work to chromosomally integrate the *csc* cassette was successful in generating a genetically stable strain (see Additional file 2: Text), as demonstrated throughout the extent of work in the study. This is in contrast to previously published plasmid expressions that have been shown to lead to complications in industrial production due to requirements for the addition of antibiotics and the burden of maintaining high-copy numbers plasmids [15, 48].

The key mutations identified in this study can be used as engineering parts for strain engineering. Given that a similar set of mutations enables enhanced growth on both glucose and sucrose, it is reasonable to expect that such mutations and strains have growth benefits on similar substrates, or mixes of such substrates, and this represents an avenue forward for generating a more universally applicable platform strain [34, 36, 49]. Previously, the key mutations identified in the RNA polymerase subunits (RNAP) *rpoB* and *rpoC* were found to have large-scale systematic transcriptional changes that influence specific cellular processes and to be responsible for fast growth [34, 36] on minimal medium. The benefits of such mutations in bioprocessing have been demonstrated and establish how strains with such mutations can serve as platform strains for enhanced production phenotypes [50]. Consequently, it appears that the fast-growing phenotype of K-12 on sucrose is more likely more due to a systems level regulatory change rather than changes specific to sucrose utilization.

There are a number of avenues to explore to build off of the results from this study. Such work could include an effort to study the impact of the key mutations individually, or in combination, on the fast growth phenotype. One interesting feature to pursue was that the evolved K-12 strains demonstrated lower SURs on sucrose than the W strain and this could be further examined. It has been shown that *E. coli* W maintains a highly oxidative metabolic state either on glucose or sucrose with low accumulation of overflow metabolites such as acetate [51]. The two *E. coli* strains possess significantly different genome sizes (4.90 Mbp for W vs 4.64 Mbp for K-12) with different numbers of genes, pseudogenes, and mobile elements [10], therefore a comparative genomic study to associate this particular feature was not apparent. A potentially fruitful approach to address this observed difference would likely include growth of the evolved and unevolved *E. coli* W and K-12 strains along with multiple omics assays to better understand the underlying mechanisms, as performed previously [36], in a sucrose utilizing context. Further, such an analysis with an engineered production pathway would also place the evolved phenotypes in a relevant production context.

In summary, ALE has been demonstrated to generate strains with reproducible causal mutations for increased sucrose utilization for *E. coli* under a strict growth rate selection pressure. Whole genome re-sequencing revealed the underlying causal mutations of the evolved strains. Thus, the ALE derived clones and the beneficial mutations represent a promising platform for developing a sucrose-based bioproduction chassis and can provide a good starting point to develop sucrose utilization in other industrially relevant *E. coli* strains.

## Additional files

**Additional file 1: Data file S1.** All mutation list of ALE resequenced clones and populations.

**Additional file 2: Text file S1.** Full strains design description and different genetic manipulation methods.

## Data Availability

All data generated or analyzed during this study are included in this published article [and its additional files].

## Abbreviations

ALE: adaptive laboratory evolution
SUC: sucrose
SUR: sucrose uptake rate
APR: acetate production rate
GLUALE: glucose adaptive laboratory evolution
RNAP: RNA polymerase
AAR: amino acids residues
