# Supplementary material for: Generation of an E. coli platform strain for improved sucrose utilization using adaptive laboratory evolution
Source: Microb Cell Fact. 2019 Jun 29;18:116. doi: 10.1186/s12934-019-1165-2 (PMC6599523; doi:10.1186/s12934-019-1165-2)
Supplement: Supplementary file 2 — Additional file 2: Text file S1. Full strains design description and different genetic manipulation methods. [file 12934_2019_1165_MOESM2_ESM.docx]

**Generation of an *E. coli* platform strain for improved sucrose utilization using adaptive laboratory evolution**

**Authors:**

Elsayed T. Mohamed^1^, Jenny Landberg^1^, Hemanshu Mundhada^1^, Isaac Cann^2^, Roderick I. Mackie^2^, Alex Toftgaard Nielsen^1^, Markus J. Herrgård^1^, Adam M. Feist^1,3^

**Corresponding Author:**

Adam M. Feist^1,2^, E. mail address; afeist@ucsd.edu

**Affiliations**

^1^Novo Nordisk Foundation Center for Biosustainability, Technical University of Denmark, Building 220, Kemitorvet 2800 Kgs. Lyngby, Denmark

^2^Department of Animal Sciences, Institute for Genomic Biology and Energy Biosciences Institute, University of Illinois, Urbana, IL 61801, USA

^3^Department of Bioengineering, University of California, 9500 Gilman Drive La Jolla, San Diego, CA 92093, USA

**Additional text, figures and tables**

1. **K-12 strains design for sucrose utilization (SUC^+^)**

Plasmids used in the current study are listed in Table 1. *E. coli* DH5⍺ was used for molecular cloning and plasmid preparations. Sucrose utilizing strain for the ALE was constructed using K-12 MG1655 as a parent strain. Chromosomal integration of the *csc* cassette into the genome was carried out via a ‘clonetegration’ approach as described previously in the published protocol by St-Pierre et al., 2013 [1].

The coding sequence of *csc* cassette, i.e. genes *cscK*, *cscB*, *cscA*, was amplified from donor *E. coli* W using forward primer ﻿5’- ATGCATCUGGGATATAGAGCTATCGACAACAACCG G -3’ and reverse primer 5’-AGAGGGCUTTATGTTAACCCAGTAGCCAGAGTGCTC-3’. The *csc* cassette was initially assembled into the pOSIP-CH backbone using a traditional restriction digest and ligation approach resulting in the pOSIP-CH-cscBKA plasmid. The resulting cloning mixture, i.e. pOSIP-CH-cscBKA, was then transformed into electro-competent cells. The phage integrations genes and antibiotic resistance cassette, i.e. ﻿the integration module, were cured with pE-FLP as described by St-Pierre et al., 2013 as of the original protocol to yield the MGcscABK strain **Supplementary** **Figure 4**.

The MGcscABKp derivative of MGcscBKA with a single point mutation in *cscB* (Q353H), was engineered by using QuikChange® Site-Directed Mutagenesis Kit (Agilent Technologies). The *cscB* gene from the pOSIP-CH-csc plasmid from the ‘clonetegration’ step was used as the template with oligonucleotides 5’- GATCTTTCTGATTGGTTTTCACATTGCCAGTTCGCTTG-3’ as forward primer, which carried the SNP mutation, and 5’-CAAGCGAACTGGCAATGTGAAAACCAATCAGAAAGATC-3’ as reverse primer targeting the *cscB* gene. The resulting plasmid, i.e. pOSIP-CH-cscBKAp, was transformed into electro-competent K-12 MG1655 cells for clonetegration as described above.

During all the integration steps, colonies were screened on selective or non-selective plates via single colony PCR with OneTaq master mix. PCR amplifications for cloning or genomic integration were carried out using Q5 polymerase. Enzymes utilized for PCR amplification, restriction digestion and ligation were obtained from Thermo Fisher (Fermentas).

1. **Additional Tables list**

Table S1: strains and plasmids

| Strain or Plasmid | Genotype | Reference/Source |
| --- | --- | --- |
| pE-FLP | *ori*R101, *repA*101ts, Amp^R^, FLP recombinase expressed by *pE* | [1] |
| pOSIP-CH | pUC *ori*, RK6γ *ori*, Cm^R^, *attP* HK022, ccdB, HK022 integrase expressed by *λ p_r_* under control of *λ* cI857 | [1] |
| pOSIP-CH-cscBKA | pOSIP-CH cloned with the sucrose utilization pathway, *cscB*, *cscK* and *cscA* | This study |
| pOSIP-CH-cscBKAp | pOSIP-CH cloned with the sucrose utilization pathway and the permease (*cscB*) contains a point mutation. | This study |
| K-12 MG1655 | *F^-^ λ^-^ ilvG rfb-50 rph-1* | N/A |
| *E. coli* W | Wild type | DSM #1116 |
| MGcscBKA | K-12 MG1655 torS-cscBKA | This study |
| MGcscBKAp | K-12 MG1655 torS-cscBKAp | This study |

1. **Additional Figures List**

**Figure S1**: Physiological characterization of evolved E. coli W clones on sugarcane juice (SCJ). Represented are plots of the growth curves, substrate consumption and byproduct production on SCJ medium of the starting strain versus the evolved strain of E. coli W, respectively. Start strains are denoted with (*SS). Error bars were computed based on technical replicates for each strain (n=2).

**Figure S2**: Physiological characterization of MGcscBKA evolved clones on sugarcane juice (SCJ). Represented are plots of the growth curves, substrate consumption and byproduct production on SCJ medium of the starting strain versus the evolved strain for each of MGcscBKA. Start strains are denoted with (*SS). Error bars were computed based on technical replicates for each strain (n=2).

**Figure S3**: Physiological characterization of the evolved selected clones on sugarcane juice (SCJ). Represented are plots of the growth curves, substrate consumption and byproduct production on SCJ medium of the starting strain versus the evolved strain of MGcscBKAp. Start strains are denoted with (*SS). Error bars were computed based on technical replicates for each strain (n=2).

**Figure S4**: Integration location of the *csc* cassette into K-12 MG1655 chromosome. Presented is the precise location of the *csc* cassette on the genome (base pair scale) along with the flanking scar regions from the backbone plasmid on both the upstream and downstream coordinate. *Csc* gene clusters were integrated between *torT* and *torS* genes for MGcscBKA and its derivative MGcscBKAp engineered strains.


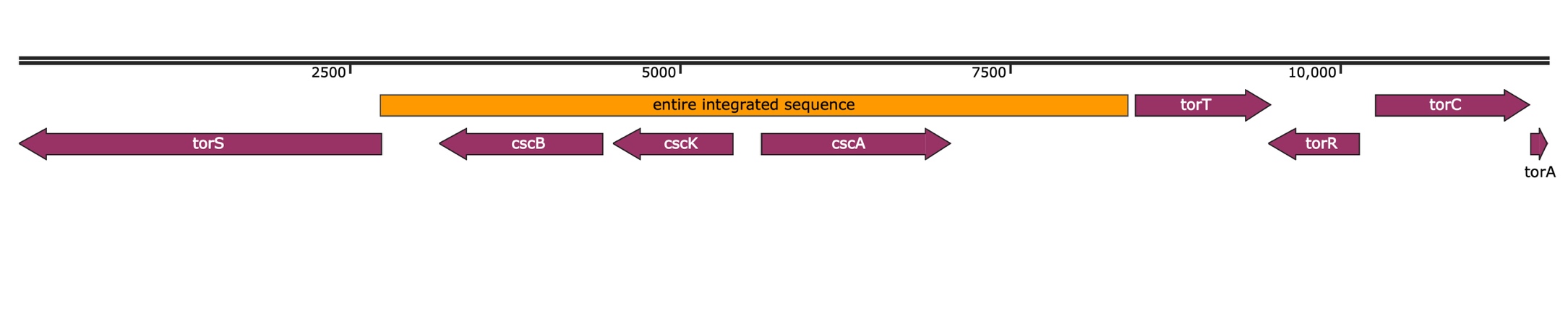


**References**

1. St-Pierre F, Cui L, Priest DG, Endy D, Dodd IB, Shearwin KE. One-Step Cloning and Chromosomal Integration of DNA. ACS Synth Biol [Internet]. American Chemical Society; 2013 [cited 2019 Jun 4];2:537–41. Available from: http://pubs.acs.org/doi/10.1021/sb400021j
